# Th17-related cytokines and chemokines are associated with immune dysregulation and severity in schizophrenia

**DOI:** 10.3389/fpsyt.2025.1675129

**Published:** 2025-10-22

**Authors:** Han Liang, Jing Xu

**Affiliations:** ^1^ Department of Clinical Laboratory, The Second Affiliated Hospital of Dalian Medical University, Dalian, Liaoning, China; ^2^ Department of Clinical Laboratory, Huai’an Hospital, Huaian, China

**Keywords:** schizophrenia, cytokines, chemokines, Th17 pathway, neuroinflammation, IL-17, CCL20, immune dysregulation

## Abstract

**Background:**

Schizophrenia is a complex neuropsychiatric disorder increasingly recognized as involving neuroimmune dysregulation. Among immune pathways, the Th17 axis and chemokine-mediated signaling have gained attention for their roles in chronic inflammation and neurotoxicity.

**Objectives:**

This study aimed to examine serum levels of Th17-related cytokines (IL-17, IL-21, IL-22, IL-23) and chemokines (CCL2, CCL5, CCL20, CXCL10, IL-8) in schizophrenia patients compared to healthy controls and to explore their associations with symptom severity and laboratory parameters.

**Methods:**

A total of 77 schizophrenia patients and 41 healthy controls were assessed. We used ELISA to find out how much cytokines and chemokines were in the serum. The Positive and Negative Syndrome Scale (PANSS) was used to rate how bad the symptoms were. We used Mann–Whitney U tests, Spearman correlations and principal component analysis (PCA) to look at the data.

**Results:**

Patients had much greater levels of IL-17, IL-22, IL-8and CCL20 (p < 0.001), as well as higher levels of CXCL10 and CCL5 and lower levels of CCL2. IL-17, IL-21, IL-22and CCL2 all had positive relationships with the intensity of negative symptoms (N-PANSS). PCA showed that immunological markers grouped in different ways, with IL-21/IL-23 and IL-17/IL-8 being the most important inflammatory parts.

**Conclusion:**

Our results suggest that Th17-related inflammation plays a key role in schizophrenia, especially when it comes to negative symptoms. Immune marker profiling may help us understand how diseases work and find subgroups that may be treated in a more tailored way.

## 1 Introduction

Schizophrenia is a multifaceted neuropsychiatric disorder, marked by impairments in cognition, communication, perception, will and behavior (1). Schizophrenia impacts around 1% of the global population, whereas approximately 3% may encounter psychosis at some point in their lives (2), resulting in considerable demands on healthcare, society and the economy (3). While the exact molecular foundation of schizophrenia remains unclear, there is now data suggesting that schizophrenia is a neuroimmune condition (4–7).

In the 1990s, the macrophage-T-lymphocyte theory, the first comprehensive theory of schizophrenia, was published (8). This idea posits that early neurodevelopmental abnormalities resulting from intrauterine infections may predispose individuals to subsequent injuries that activate immune-inflammatory and nitro-oxidative stress pathways. Subsequently, an increasing body of evidence indicates that immune-inflammatory mechanisms in the peripheral circulation and central nervous system (CNS) contribute to the genesis of schizophrenia, predominantly via systemic inflammation leading to microglial activation (9–13). Schizophrenia is characterized by the activation of the immune-inflammatory response system (IRS), driven by activated M1 macrophages and T helper (Th)1 and Th17 cells, as well as the activation of the compensatory immune-regulatory system (CIRS), which downregulates the primary IRS to prevent hyperinflammation (12, 14). Furthermore, heightened neurotoxicity resulting from elevated concentrations of toxic cytokines (e.g., IL-1β, IL-6, IL-8, TNF-αand IFN-γ) and chemokines (e.g., CCL11, CCL2, CXCL8 and CXCL10) is markedly correlated with the schizophrenia phenome (15). Significant increases in neuroimmunotoxic pathways are notable in Schizophrenia, along with reduced CIRS protection and an enhanced phenome (16, 17).

Inflammatory cytokines and their regulatory factors are relatively big molecules that cannot traverse the blood-brain barrier under normal physiological circumstances (18). Chemokines, a category of mediators, are essential in neuroinflammation by recruiting peripheral leukocytes to the central nervous system. Molecules including CCL20, CXCL10, CCL2and CCL5 are pivotal in this process, each facilitating the recruitment of distinct immune cell subsets: Th17 cells (CCL20), Th1 and NK cells (CXCL10), monocytes (CCL2) and T lymphocytes (CCL5). These chemokines are increased in response to inflammatory stimuli and are synthesized by activated glial cells, including microglia (19–22). Their effects not only regulate T cell activity but also enhance local immunological responses inside the brain tissue. The participation of these chemokines in several inflammation-associated ailments, including rheumatoid arthritis and various mental disorders, indicates their possible relevance in the neuroinflammatory and neuroprogressive pathways that underlie schizophrenia and similar illnesses (23).

Furthermore, recent years have seen heightened focus on the intricate relationship between immune-inflammatory pathways and metabolic abnormalities in mental diseases like schizophrenia (24). Chemokines and cytokines, particularly those associated with Th17 responses, contribute to immune cell recruitment and may potentially affect systemic metabolic conditions (25). Metabolic metrics, including fasting blood glucose (FBS), lipid profile components (such as LDL, HDL and triglycerides) and body mass index (BMI), serve as essential markers of energy balance and inflammatory load (26–28). Evidence indicates that immune-mediated inflammation may play a role in the metabolic abnormalities associated with schizophrenia (19). In a Chinese study of first-episode, drug-naïve schizophrenia patients, the incidence of metabolic syndrome was found at 10.93%, markedly above that of matched healthy controls (29).

Although the immune-inflammatory and metabolic components of schizophrenia are increasingly recognized, few studies have studied the interaction between circulating cytokines and chemokines, metabolic parameters and clinical symptomatology. Most research has examined immunological or metabolic indicators in chronic or treated patients. Thus, integrated studies of immune-inflammatory profiles and metabolic state in connection to clinical severity are few, especially in schizophrenia populations where systemic immune activation may contribute to disease progression and symptom heterogeneity. The present study compares the serum levels of selected cytokines and chemokines—IL-17, IL-22, IL-23, IL-8, IL-21, CCL20, CXCL10, CCL2and CCL5—in schizophrenia patients to healthy controls due to growing evidence linking immune-inflammatory and metabolic dysregulation to schizophrenia. We also examine the relationships between these immunological markers and metabolic measures such as fasting blood glucose, lipid profile, body mass index and psychometric scores like PANSS. This study integrates immunological, metabolic and clinical domains to understand neuroinflammation and metabolic imbalance in schizophrenia better and find new biomarkers implicated in its development and clinical heterogeneity.

## 2 Materials and methods

### 2.1 Study design and participants

This case–control study was conducted in the Second Affiliated Hospital of Dalian Medical University between May 2024 and October 2024. Patients with schizophrenia were recruited from psychiatric wards, while healthy control participants were enrolled via advertisements posted on hospital notice boards and distributed online throughout the city. All participants provided written informed consent after being fully informed of the study objectives and procedures. The protocol of this study was reviewed and approved by the local ethical committee from The Second Affiliated Hospital of Dalian Medical University (KY2024-482-01).

The study included 77 patients with schizophrenia and 41 healthy controls. The sample size was based on feasibility within the recruitment period and is comparable to previously published psychoneuroimmunology studies that successfully identified cytokine alterations in schizophrenia using similar cohort sizes (30).

Out of the total screened participants, 77 patients diagnosed with schizophrenia and 41 healthy controls were included. A board-certified psychiatrist confirmed the diagnosis based on DSM-5 criteria. Control participants were screened to ensure the absence of any major psychiatric disorders. Both groups were matched by age, sex, and BMI to reduce the risk of demographic confounding.

All participants were inpatients admitted with a primary diagnosis of schizophrenia. Patients with other major psychiatric or medical disorders, or those receiving immunomodulatory or non-antipsychotic psychotropic medications, were excluded to minimize confounding effects. Symptom severity was assessed at the time of blood sampling using the Positive and Negative Syndrome Scale (PANSS).

### 2.2 Clinical assessment

We used the PANSS, which has 30 questions about positive and negative symptoms as well as general psychopathology, to rate how bad the schizophrenia group’s symptoms were. The total score, which is based on a 7-point scale, gives a good idea of how bad the illness is overall. The tool has a Cronbach’s alpha of 0.83 which means it is reliable for psychometrics (31, 32).

### 2.3 Blood collection and immune marker quantification

Participants were instructed to avoid caffeine intake and strenuous physical activity for at least 30 minutes prior to venipuncture. All samples were collected between 09:00 and 11:00 A.M. after an overnight fast. For inpatients, venous blood was drawn within the first 24 hours of hospital admission and before the administration of antipsychotic medication. Approximately 3 mL of venous blood was drawn into serum separator tubes, allowed to clot at room temperature for 30 minutes, and centrifuged at 1,500 × g for 10 minutes at 4°C. Serum was aliquoted and stored at −80°C until assay, avoiding repeated freeze–thaw cycles.

Serum concentrations of IL-17A, IL-21, IL-22, IL-23, IL-8 (CXCL8), CCL2, CCL5, CCL20, and CXCL10 were measured using commercially available ELISA kits (IL-17A, IL-21, IL-22, and IL-23: Mabtech; all other analytes: BioLegend) following the manufacturers’ instructions. The detection ranges and limits of detection (LOD) for each analyte were as follows: IL-17A, IL-21, IL-22, and IL-23: 3.9–1000 pg/mL (LOD 3.9 pg/mL); IL-8: 31.2–2000 pg/mL (LOD 31.2 pg/mL); CCL20: 2.5–160 pg/mL (LOD 2.5 pg/mL); CXCL10: 15.6–1000 pg/mL (LOD 15.6 pg/mL); CCL5: 7.8–500 pg/mL (LOD 7.8 pg/mL); and CCL2: 7.8–500 pg/mL (LOD 7.8 pg/mL). All samples were assayed in duplicate. The intra- and inter-assay coefficients of variation (CVs) for each analyte were as follows: IL-17A (intra 2.8%, inter 3.6%), IL-21 (intra 3.2%, inter 4.1%), IL-22 (intra 2.9%, inter 4.3%), IL-23 (intra 3.5%, inter 5.0%), IL-8 (intra 4.2%, inter 5.6%), CCL20 (intra 2.7%, inter 3.9%), CXCL10 (intra 3.4%, inter 4.6%), CCL5 (intra 2.5%, inter 3.8%), and CCL2 (intra 3.1%, inter 4.5%). Hemolyzed or lipemic samples were excluded. Outliers defined as >3 SD above the group mean were re-assayed, and confirmed extreme values were retained but considered in sensitivity analyses.

### 2.4 Statistical analysis

All data were analyzed using SPSS version 27 (IBM Corp., Armonk, NY). Descriptive statistics were presented as median with interquartile range (IQR). Normality of data distributions was tested using the Shapiro–Wilk test. All cytokine and chemokine variables, except for CCL5 (W = 0.98, p = 0.10), significantly deviated from normality. Consequently, the Mann–Whitney U test was used for group comparisons. For CCL5, we additionally performed a parametric Welch’s t-test. Correlation analyses were performed using Spearman’s rank correlation to assess associations between immune markers and clinical/laboratory variables. A principal component analysis (PCA) with Varimax rotation was used to extract clustering patterns among immune markers. A two-sided p-value < 0.05 was considered statistically significant.

## 3 Results

### 3.1 Demographic and clinical characteristics

A total of 118 people took part in the study, including 77 schizophrenia patients and 41 healthy controls. We could only get demographic and clinical information about the patient group. The patients were between 20 and 65 years old, with an average age of 41.08 years (SD = 9.77). Among them, 74.3% were male and 25.7% were female.

Laboratory results showed a wide range of values. The mean white blood cell (WBC) count was 7.69 ×10³/μL (SD = 2.36) and the mean hemoglobin (Hb) level was 12.58 g/dL (SD = 1.45). The mean body mass index (BMI) was 23.32 (SD = 7.05). Inflammatory indicators varied substantially, with a mean C-reactive protein (CRP) level of 13.27 mg/L (SD = 46.99) and an erythrocyte sedimentation rate (ESR) of 18.26 mm/hr (SD = 16.58) (**Table 1**).

**Table 1:** Demographic, clinical, and laboratory characteristics of patients.

Parameters	Patients	Normal range (Unit)
Age *Mean (SD)*	41.08 (9.77)	(Year)
BMI *Mean (SD)*	23.32 (7.04)	18.5 to 24.9 (kg/m²)
WBC *Mean (SD)*	7.686 (2.3637)	4.5–11.0 ×10^9^ (/L)
RBC *Mean (SD)*	4.4249 (0.48211)	4.3–5.9 ×10¹²/L (men), 3.8–5.2 ×10¹²/L (women)
Hb *Mean (SD)*	12.581 (1.449)	13.5–17.5 g/dL (men), 12.0–16.0 g/dL (women)
PLT *Mean (SD)*	243.064 (84.3266)	150–400 ×10^9^/L
AST *Mean (SD)*	26.49 (20.928)	8–40 U/L
ALT *Mean (SD)*	22.73 (17.194)	7–56 U/L
ALKP *Mean (SD)*	202.27 (67.016)	8–40 (U/L)
TSH *Mean (SD)*	2.611 (2.5793)	0.4–4.0 mIU/L
FT4 *Mean (SD)*	8.582 (1.6362)	0.8–1.8 ng/dL
FT3 *Mean (SD)*	1.161 (0.2763)	2.3–4.1 pg/mL
BUN *Mean (SD)*	14.69 (4.372)	0.8–1.8 (ng/dL)
Cr *Mean (SD)*	0.977 (0.2083)	0.7–1.3 mg/dL (60–110 µmol/L)
CPK *Mean (SD)*	288.908 (509.9544)	3.0–7.0 mmol/L (8–20 mg/dL)
LDH *Mean (SD)*	347.69 (136.56)	60–118 µmol/L (0.7–1.3 mg/dL)
FBS *Mean (SD)*	126.75 (101.628)	70–110 mg/dL (3.8–6.0 mmol/L)
Bill T *Mean (SD)*	0.883 (0.657)	50–150 (U/L)
Bill D *Mean (SD)*	0.251 (0.142)	3.8–6.0 mmol/L (70–110 mg/dL)
Na *Mean (SD)*	139.91 (4.237)	1–17 µmol/L (0.1–1.0 mg/dL)
K *Mean (SD)*	4.114 (0.4167)	0–5 µmol/L (0–0.3 mg/dL)
P *Mean (SD)*	4.004 (1.1052)	135–145 (mmol/L)
Ca *Mean (SD)*	9.327 (0.9553)	3.5–5.0 (mmol/L)
ESR *Mean (SD)*	18.26 (16.583)	<15 mm/hr (men), <20 mm/hr (women)
CRP *Mean (SD)*	13.27 (46.9983)	<5 mg/L
TG *Mean (SD)*	127.96 (83.2)	0.7–1.1 (mmol/L)
CHOL *Mean (SD)*	178.75 (49.825)	Males: <15, Females: <20 (mm/hr)
LDL *Mean (SD)*	99.87 (25.015)	<5 (mg/L)
HDL *Mean (SD)*	44.09 (11.476)	<1.7 mmol/L (150 mg/dL)
Mg *Mean (SD)*	2.0996 (0.23795)	<5.2 mmol/L (200 mg/dL)
lym *Mean (SD)*	31 (10.339)	<3.4 mmol/L (130 mg/dL)
neutr *Mean (SD)*	63.9 (11.153)	>1.0 (men), >1.3 (women) (mmol/L)
ECT *N (%)*	47 (38.8)	None
*History of Hospitalization N (%)*	67 (83.8)	None
Smoking *N (%)*	42 (52.5)	None
Suicidal Ideation *N (%)*	10 (12.5)	None
Suicide Attempt *N (%)*	16 (20)	None

### 3.2 Comparison of cytokine and chemokine levels

**Figure 1:**
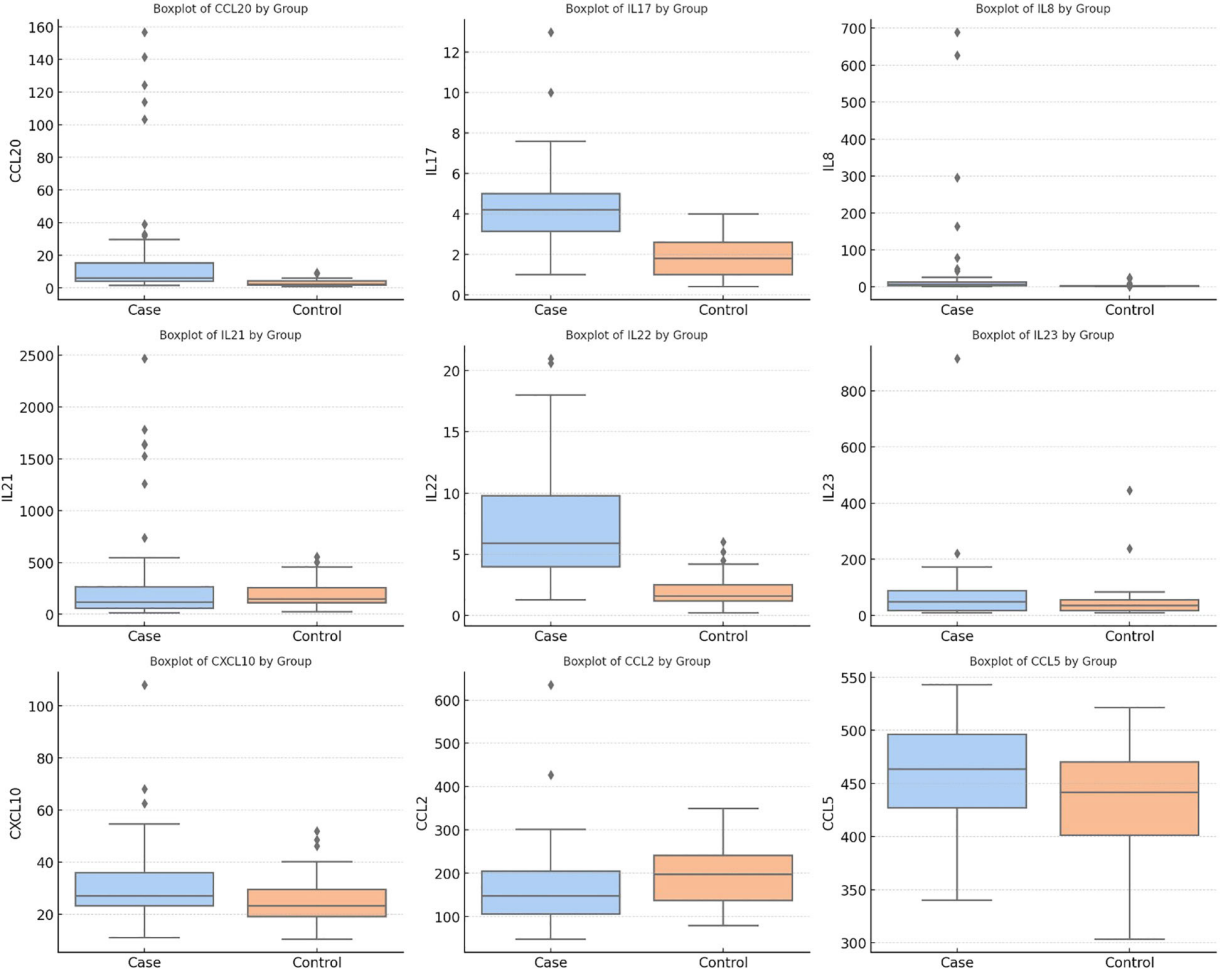
Serum levels of cytokines and chemokines in schizophrenia patients and healthy controls.

We used the Mann–Whitney U test to look at the differences in cytokine and chemokine levels between patients and controls. The patient group had statistically significant increases in CCL20, IL-17, IL-8, CCL2 and IL-22 (all p < 0.001). Additionally, patients exhibited higher levels of CXCL10 (p = 0.028) and CCL5 (p = 0.03). In contrast, the schizophrenia group had a much lower level of CCL2 than the control group (p < 0.001). There were no significant differences between IL-21 (p = 0.06) and IL-23 (p = 0.329) (**Table 2**, **Figure 1**).

**Table 2:** Group comparisons of serum cytokine and chemokine levels between schizophrenia patients and healthy controls.

Marker	Case median (IQR)	Control median (IQR)	p-value (2-tailed)
CCL20	6.4 (15.2-4.1)	2.5 (5-1.8)	<0.001
IL17	4.1 (5-2.4)	1.8 (2.6-1)	<0.001
IL8	6.6 (13.375-3.05)	2.0 (3.1-2)	<0.001
IL21	97.5 (228.5-174.27)	150.0 (255-100)	0.06
IL22	6.0 (10.07-4.25)	1.7 (3.6-1.2)	<0.001
IL23	47.6 (87-17)	35.1 (57.15-17.3)	0.329
CXCL10	27.0 (33.50-22.95)	23.6 (34.6-19.4)	0.028
CCL2	144.9 (205.40-102.90)	198.0 (241-131.7)	<0.001
CCL5	462.4 (494.97-426.25)	437.0 (468.5-402.6)	0.03

### 3.3 Correlation analysis among immune markers

**Figure 2:**
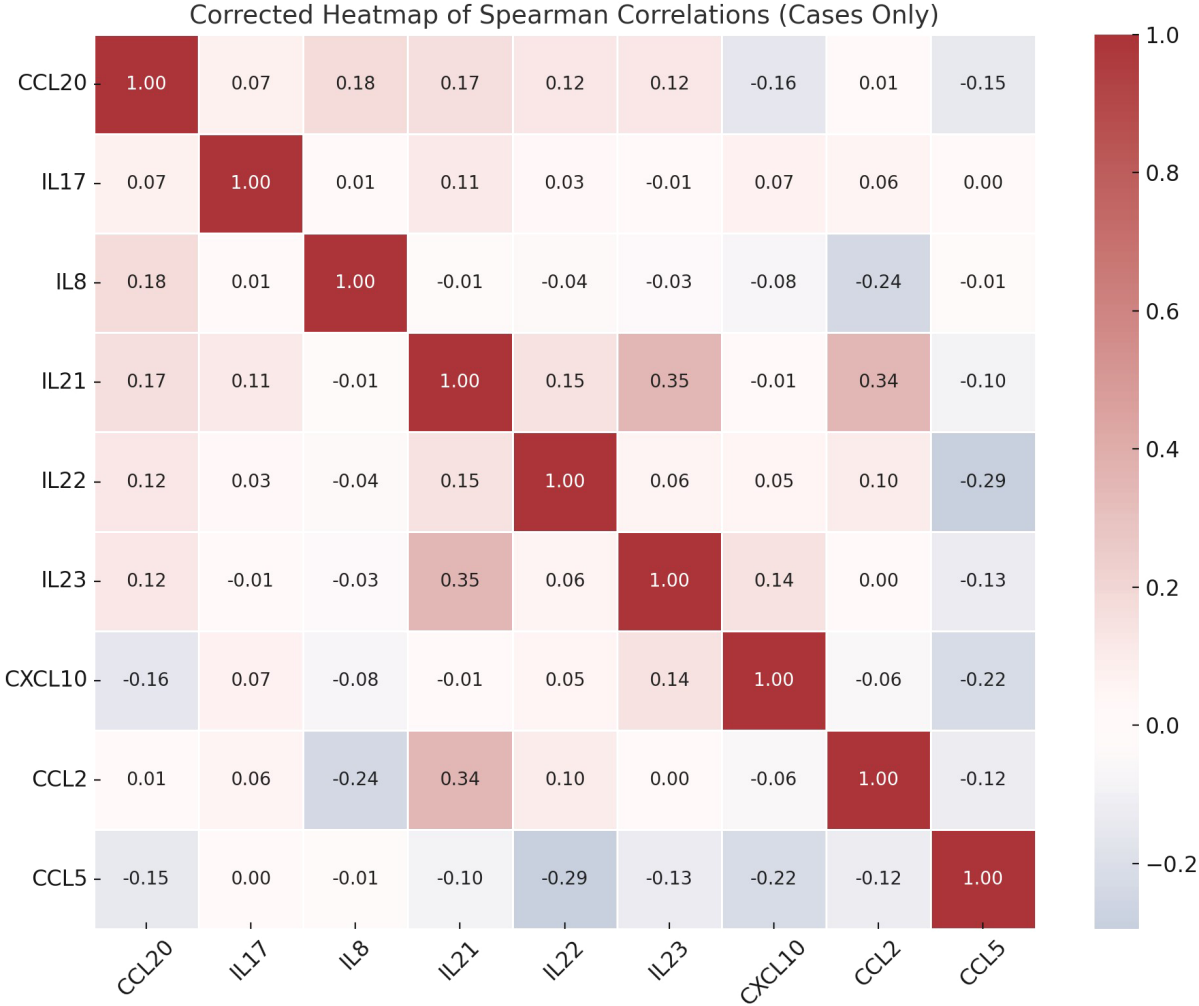
Spearman correlation heatmap of immune markers in schizophrenia patients.

Correlation analysis showed that there were several statistically significant links between the immune markers that were measured. IL-8 and CCL2 were positively correlated (p = 0.039) and IL-21 was significantly linked to both IL-23 (p = 0.002) and CCL2 (p = 0.003). A significant correlation was also observed between IL-22 and CCL5 (p = 0.015) (**Figure 2**).

### 3.4 PANSS association analysis

Clinical symptom severity was assessed using the Positive and Negative Syndrome Scale (PANSS). The average scores were 25.38 (SD = 4.78) for the positive subscale (P-PANSS), 31.54 (SD = 8.19) for the negative subscale (N-PANSS) and 17.77 (SD = 3.92) for the general psychopathology subscale (E-PANSS). The disorganization and depression subcomponents had means of 23.93 (SD = 3.65) and 12.13 (SD = 3.42), respectively.

**Figure 3:**
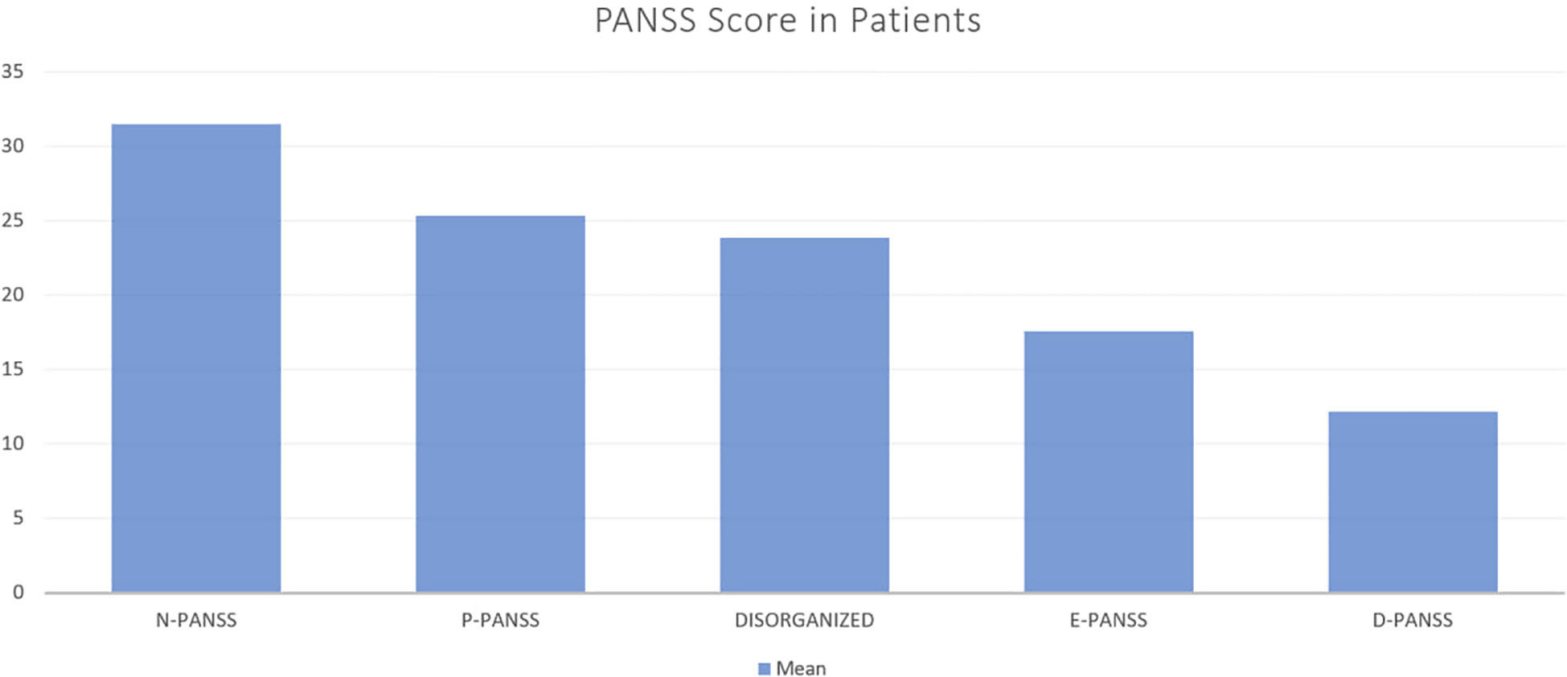
Mean PANSS Subscale Scores in Patients with Schizophrenia. This bar chart illustrates the mean scores of different PANSS (Positive and Negative Syndrome Scale) subscales in a sample of patients. The subscales include Negative symptoms (N-PANSS), Positive symptoms (P-PANSS), Disorganized thought (DISORGANIZED), Excitement (E-PANSS), and Depression (D-PANSS).

The relationship between immune marker levels and the severity of negative symptoms in schizophrenia was evaluated using correlation analysis with the N-PANSS subscale. Statistically significant but weak positive correlations were observed between N-PANSS scores and IL-17 (r = 0.24, 95% CI [0.01, 0.44], p = 0.044), IL-21 (r = 0.25, 95% CI [0.01, 0.45], p = 0.036), IL-22 (r = 0.23, 95% CI [0.00, 0.43], p = 0.050), and CCL2 (r = 0.27, 95% CI [0.04, 0.47], p = 0.020) (**Figure 3**).

### 3.5 Principal component analysis

**Figure 4:**
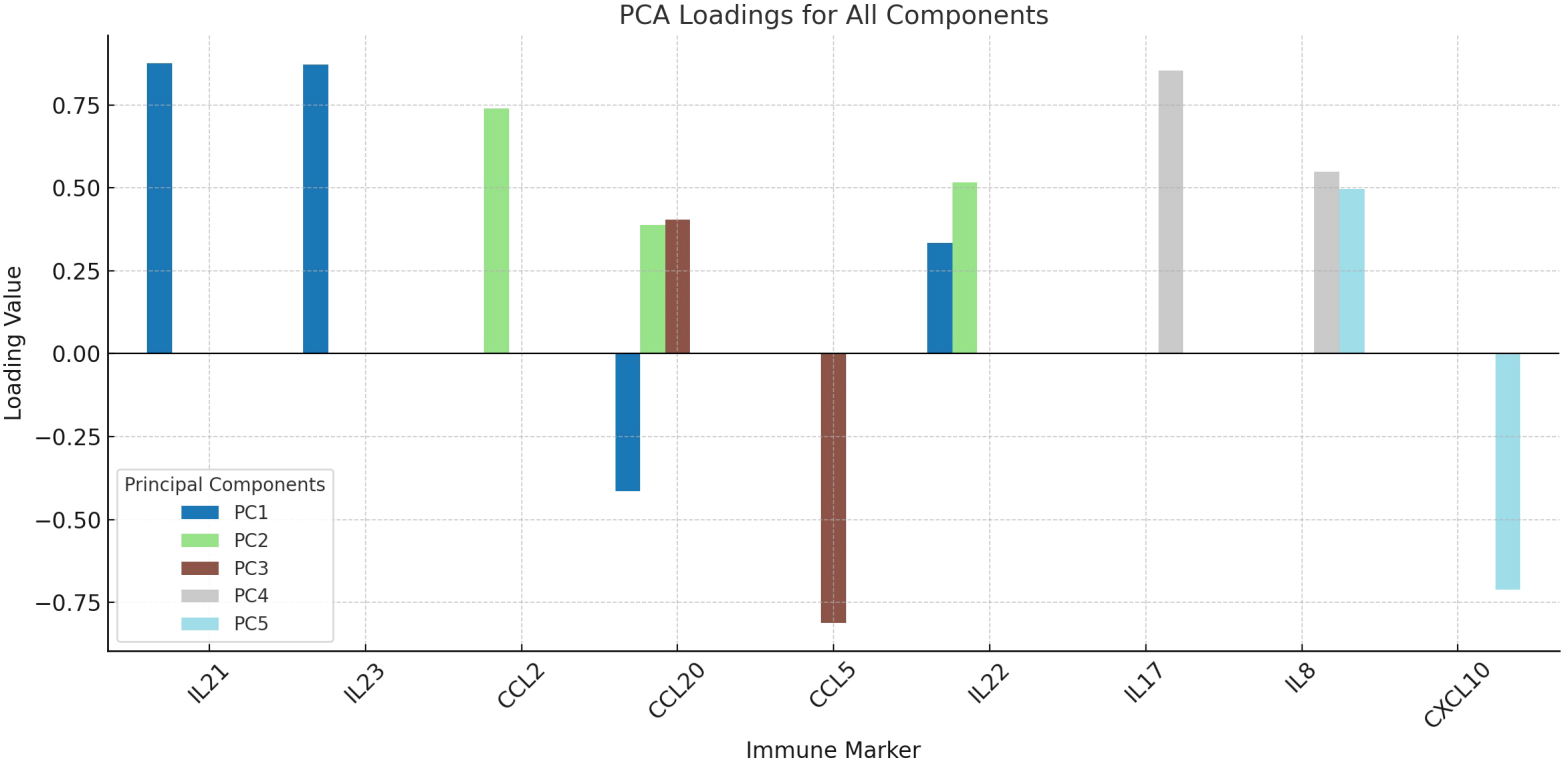
Principal component analysis (PCA) of inflammatory markers in schizophrenia. The loading plot summarizes the contribution of each immune marker to five principal components derived from PCA with Varimax rotation. IL-21 and IL-23 loaded strongly on PC1, reflecting Th17 axis activation, while IL-17 and IL-8 loaded on PC4, indicating neutrophil-related inflammation. This dimensionality reduction highlights distinct immunological subdomains among patients.

We used PCA to look at the underlying structure of cytokine and chemokine profiles. The data were used to find five main components. The first component had high loadings for IL-21 and IL-23 (0.876 and 0.872, respectively). D-PANSS and CCL2 were the second component, with loadings of 0.762 and 0.740. CCL20 made moderate contributions to the first (−0.415), second (0.388) and third (0.404) components. CCL5 had a strong negative effect on the third component (−0.812), while IL-22 had effects on both the first (0.334) and second (0.516) components. IL-17 had a strong load on the fourth component (0.854) and IL-8 had strong loads on the fourth (0.548) and fifth (0.497) components. CXCL10 had a strong negative loading on the fifth component (−0.712) (**Table 3**, **Figure 4**).

**Table 3:** Rotated component matrix of principal component analysis (PCA) for Th17-related cytokines and chemokines.

Analyzed factors	Components				
	PC1	PC2	PC3	PC4	PC5
SMEAN (IL21)	0.876				
SMEAN (IL23)	0.872				
SMEAN (CCl2)		0.740			
SMEAN (CCL20)		-0.415	0.388	0.404	
SMEAN (CCL5)			-0.812		
SMEAN (IL22)		0.334	0.516		
SMEAN (IL17)				0.854	
SMEAN (IL8)				0.548	0.497
SMEAN (CXCL10)					-0.712

This table displays the rotated factor loadings derived from PCA with Varimax rotation, summarizing the contribution of each immune marker to the five principal components. IL-21 and IL-23 showed strong loadings on Component 1, suggesting a dominant Th17-related inflammatory profile, while other markers clustered around distinct components indicating heterogeneous immune activation patterns.

## 4 Discussion

### 4.1 Summary of main findings

In this case-control study, we looked at the levels of certain cytokines and chemokines in the blood of schizophrenia patients and compared them to healthy controls. We also looked at how these levels were related to the severity of negative symptoms. Our research showed that patients with schizophrenia had much higher levels of certain immune markers, such as IL-17, IL-8, IL-22and CCL20. The patient group also had higher levels of CXCL10 and CCL5, but CCL2 levels were much lower.

Correlation analysis found strong links between some immune markers, like IL-21 with IL-23 and CCL2and IL-22 with CCL5. A group of immune markers, such as IL-17, IL-21, IL-22and CCL2, were strongly linked to the N-PANSS symptom subscale. This suggests that the severity of symptoms may have an immunological basis. Also, principal component analysis (PCA) showed that there were different immunological patterns, with Th17-related cytokines and chemokines clustering together in a few components. These findings suggest a complex and potentially coordinated immune dysregulation in schizophrenia, particularly related to the Th17 pathway and associated chemotactic signals.

### 4.2 Dysregulation of Th17-related cytokines in schizophrenia

Th17 cells represent a distinct lineage of CD4^+^ T lymphocytes, primarily characterized by secretion of IL-17A, IL-17F, IL-21, IL-22 and granulocyte-macrophage colony-stimulating factor (GM-CSF), under the transcriptional control of RORγt (33). These cells are crucial for safeguarding the mucosa and the host from external infections; but, when they malfunction, they can lead to chronic inflammatory and autoimmune disorders (34). Dendritic cells and macrophages produce IL-23, which is important for the differentiation, growth and stabilization of pathogenic Th17 cells by activating the STAT3 pathway (33). IL-17 is a Th17 cytokine that acts on epithelial and endothelial cells to induce the production of chemokines such as IL-8, CCL20, and MCP-1. This facilitates the influx of neutrophils and exacerbates inflammation (18, 35). IL-22, which is often found with IL-17, mainly affects non-hematopoietic cells to improve tissue repair, produce antimicrobial peptides and keep barriers strong through STAT3 signaling (36). IL-21 functions as both an effector and an autocrine enhancer within the Th17 axis. It enhances the differentiation of Th17 cells and stimulates lymphocyte proliferation through JAK-STAT activation (37). The IL-6/IL-23/Th17 axis has been linked to neuroinflammatory processes and neuropsychiatric conditions, such as schizophrenia, by mediating neuroimmune crosstalk and possibly throwing off the balance of the blood-brain barrier (38).

In this study, plasma IL-17 levels were much higher in schizophrenia patients. IL-17 is a cytokine that induces inflammation and is mostly produced by Th17 cells. It functions by binding to the IL-17 receptor on epithelial and endothelial cells, activating the NF-κB and MAPK signaling pathways, and inducing the release of chemokines such as IL-8, CCL20, and MCP-1 (39). These chemicals help bring in neutrophils and make local inflammation worse (37). Several autoimmune and neuroinflammatory diseases have been linked to IL-17 and schizophrenia patients have been found to have higher levels of it (38). This may connect peripheral immune activation to problems in the central nervous system. Meta-analyses suggest that IL-17 and IL-23 may be trait markers that are not affected by short-term antipsychotic treatment (11). Thus, the elevation of IL-17 in schizophrenia patients may result from prolonged neuroimmune activation and IL-17’s potential involvement in compromising the blood-brain barrier or promoting glial activation.

Compared to controls, schizophrenia patients had much higher levels of IL-22 in this study. IL-22 is another cytokine produced by Th17 cells, and it is often found alongside IL-17 (36). IL-22 mostly affects non-hematopoietic cells like neurons and epithelial cells, which is different from IL-17 (40). IL-22 activates STAT3, which leads to the production of antimicrobial peptides, the regeneration of tissue and the maintenance of barrier function. But when there is long-term inflammation, IL-22 may make things worse by making pro-inflammatory cytokines like IL-6 and TNF-α more common (41). Some studies show that higher levels of IL-22 protect neurons (for example, by increasing BDNF levels) (38), while other studies show that it helps keep neuroinflammation going (42). We may have found either a protective response that compensates for damage or a sign of ongoing inflammation in epithelial or neural interfaces. IL-22 has both protective and harmful effects, which is why it needs to be studied more about schizophrenia.

Furthermore, our study found that IL-21 levels were not significantly different between groups (p = 0.065), but they were strongly related to IL-23 and CCL2. IL-21 is a cytokine that has many effects and is very important for boosting Th17 responses (43). Th17 cells secrete it, which functions in an autocrine manner to enhance their differentiation and activity by activating STAT3 (44). IL-21 also affects the growth of B-cells, the activity of NK cells and the ability of CD8+ T-cells to kill other cells (45). Results indicate that IL-21 may participate in feedback mechanisms regulating Th17-mediated immune responses. These findings suggest that IL-21 is not an immediate indicator of illness but rather a regulator of persistent immune activation.

Also, our study found that IL-23 levels were not very different between patients and controls. IL-23 is an upstream cytokine that is necessary for the stability and growth of harmful Th17 cells (46). IL-23 is made by dendritic cells and macrophages (46). It facilitates the survival and efficient functioning of Th17 cells by transmitting signals via STAT3 (38). It is part of the IL-23/IL-17 axis, which is often linked to chronic inflammatory diseases (47). Meta-analytic evidence shows that IL-23 levels stay the same during short-term antipsychotic treatment, which supports its trait-like behavior (11). The insignificance of our results may be attributed to the transient expression of IL-23, which may not have been detected at the time of sampling, or to the localized action of IL-23 and IL-21, which may not be secreted into the bloodstream. Additionally, a regulatory consumption effect during prolonged immune activation could also be a contributing factor.

While Th17-related cytokines (e.g., IL-17/IL-22) can promote neuroinflammatory processes, they also mediate host defense (e.g., induction of antimicrobial peptides). Our cross-sectional design cannot determine whether Th17 skewing is a cause or a consequence of schizophrenia. Two non-mutually exclusive models may operate: (i) an immune-to-brain pathway, whereby persistent Th17 activity perturbs blood–brain barrier integrity and glial function; and (ii) a brain-to-immune pathway, whereby longstanding negative symptoms and psychosocial stress enhance peripheral inflammation. Longitudinal sampling and interventional studies are warranted to resolve directionality.

### 4.3 Dysregulation of chemokines in schizophrenia

Chemokines are a group of small (8–10 kDa) secreted cytokine-like proteins that control the movement, activation and targeting of different types of leukocytes by binding to G protein–coupled chemokine receptors. They are very important for immune surveillance, inflammation, tissue repair and homeostasis (48). There are more than 50 chemokine ligands and almost 20 receptors, but because of practical limitations in clinical immunophenotyping, we chose to focus on five important pro-inflammatory and homeostatic chemokines that were relevant to our hypothesis: IL-8 (CXCL8), CCL2, CCL5 (RANTES), CCL20 and CXCL10. These chemokines were selected due to their established involvement in neuroinflammation, where they facilitate the recruitment of monocytes, T cells, and neutrophils. Furthermore, they have been previously linked to neuropsychiatric and inflammatory disorders.

Our data show that CCL20 levels were much higher in patients than in healthy controls. CCL20, also known as macrophage inflammatory protein-3 alpha (MIP-3α), is a chemokine that plays a key role in guiding the movement of CCR6-expressing cells, especially Th17 lymphocytes, to places where there is inflammation (49). As mentioned in the last section, Th17 dysregulation has been linked more and more to the causes of schizophrenia. Ghasemi et al. found that patients with schizophrenia and bipolar disorder had much higher levels of CCL20 in their blood, especially when looking at IL-17 and IL-6 levels. This suggests that CCL20 may be a functional link between peripheral inflammation and symptom severity (19). In addition, experimental studies in autoimmune and neuroinflammatory models have shown that blocking CCL20 lowers Th17 infiltration and inflammation, which further shows how it helps diseases spread (49, 50). Our results, along with other evidence from the literature, support the idea that CCL20 is a key chemotactic driver in Th17-related neuroimmune activation in schizophrenia and could be a good target for immunomodulation.

Compared to controls, our study found that schizophrenia patients had much higher levels of IL-8. Neutrophils are important effector cells in the innate immune response and several chemokines, the most important of which is IL-8, help bring them to sites of inflammation (51). This CXC chemokine, which is made by macrophages, endothelial cells and astrocytes, binds to CXCR1/CXCR2 receptors on neutrophils (51, 52). This causes strong chemotaxis, degranulation and adhesion molecule expression, which makes acute inflammatory responses stronger (53). In schizophrenia, high levels of IL-8 may indicate peripheral neuroimmune crosstalk. IL-17 is known to increase IL-8 production through NF-κB/MAPK pathways, which may connect Th17 activation to inflammation caused by neutrophils (54). This study findings indicate that serum IL-8 levels are elevated in individuals with schizophrenia and correlations with baseline serum IL-2 or IL-8 concentrations and therapeutic effectiveness have been identified (55). Patients diagnosed with paranoid schizophrenia had statistically significant increases in serum IL-8 levels as compared to the control group (56, 57). These similar pieces of information show how important IL-8 is as a link between Th17-driven cytokine release and neutrophil-driven inflammation in schizophrenia. Because it has known effects on the nervous system and blood vessels and may be able to change the integrity of the blood-brain barrier, high levels of IL-8 may be a sign of and contribute to ongoing neuroimmune disturbances. This makes it a possible biomarker and treatment target for psychotic disorders.

Our study found that IL-8 and CCL20 were the most important chemotactic mediators. However, the way immune responses work is like an orchestra, where even less important players like CCL2 (MCP-1), CXCL10 (IP-10) and CCL5 (RANTES) can play important roles at different stages of inflammation. Monocytes, microglia and endothelial cells produce CCL2, which brings monocytes, memory T cells and dendritic cells to inflamed tissues (58). Our results showed a big drop in circulating CCL2 levels (p = 0.002), but earlier study by Drexhage et al. found that serum CCL2 levels were higher in schizophrenia patients, especially those with metabolic syndrome. This suggests that the effects may be different for different groups of people or change over time (59). Interferon-γ causes the production of CXCL10, which is secreted by monocytes and endothelial cells (60). CXCR3 attracts activated T cells and NK cells (61). We saw a small rise, but we still don’t know exactly what it does in chronic neuroimmune signaling. Interestingly, CXCL10 levels have been different in different studies of schizophrenia. Some postmortem data show that CSF and cortical levels stayed the same (62, 63). T cells, monocytes and astrocytes all secrete CCL5, which helps T cells, eosinophils and basophils move through CCR1/3/5 binding (64). Our observations indicated a modest increase, consistent with studies of CCL5 overexpression in models of maternal immunological activation and its potential function in recruiting CD8^+^ T cells to areas of neuroinflammation (22). Overall, these results suggest that these chemokines may play bigger roles in certain stages or subtypes of immune activation in schizophrenia, even though they were not as strongly affected in our study. More long-term and subgroup analyses are needed.

### 4.4 Correlation analysis among immune markers

When we looked at how cytokines and chemokines interacted, we found several important links that could mean that the immune system is working together. IL 8 and CCL2 had a positive correlation, which means that both neutrophil and monocyte pathways were active at the same time. NF-κB controls both IL 8, a strong neutrophil chemoattractant and CCL2, which brings in monocytes and memory T cells. Both of these proteins are made in response to inflammatory signals like IL 1β and IL 17 (65). The fact that they are related suggests that there is a common activation loop in both acute and subacute inflammation.

Furthermore, we saw a strong link between IL 21 and IL 23, which further supports the involvement of the Th17 axis. IL 21 is an important autocrine amplifier of Th17 differentiation. It also increases the expression of IL 23R, which produces IL 23 more responsive through STAT3 signaling (44). IL 21 was also linked to CCL2, which suggests that there is a link between adaptive Th17 responses and the movement of innate monocytes. These connections are similar to what has been found in autoimmune diseases like lupus and psoriasis, where IL 21 helps both T-cells grow and monocytes move (43–45). Another interesting finding was the link between IL 22 and CCL5. This could mean that they both play a role in keeping tissues stable while also activating inflammatory pathways. IL 22 affects epithelial and neuronal tissues through STAT3and CCL5 brings in T cells and eosinophils through CCR5 (36, 42).

IL 17 is an important effector cytokine in the Th17 pathway, but it didn’t have a strong relationship with other immune markers that were measured. It could be because it only shows up at certain times or only works in certain parts of the body, like the CNS, which is more than what peripheral serum levels can show. IL 17’s effects may peak at times that aren’t shown in cross-sectional sampling, just like an orchestral system.

The dopaminergic system may be particularly susceptible to peripheral inflammatory signals through several mechanisms: microglial priming within striatal and mesocortical circuits, cytokine-induced shifts in kynurenine pathway metabolites that modulate dopamine synthesis and release, and receptor-mediated effects on synaptic plasticity. Region-specific vulnerability (high metabolic demand, dense microglial networks, and cytokine receptor expression) may thus explain why systemic immune activation does not uniformly affect the whole brain. In this context, serum markers should be viewed as system-level proxies that interact with circuit-level susceptibilities.

### 4.5 Correlation with negative symptom severity

Negative symptoms of schizophrenia, like lack of emotion, lack of speech, lack of pleasure, social withdrawal and lack of motivation, are long-lasting problems that make it very hard to function and have a good quality of life (66). The N-PANSS is a well-known way to measure how bad negative symptoms are (67).

We found that there were statistically significant positive relationships between N-PANSS scores and several Th17-related markers, including IL 17, IL 21, IL 22 and the chemokine CCL2. These connections suggest that activating the Th17 axis in the periphery and monocyte-involved chemotaxis may be linked to worse negative symptoms. IL-17 and IL-22 can alter glial cell function, disrupt dopaminergic and glutamatergic neurotransmission in cortico-striatal circuits, and potentially diminish motivation and affect, which are two primary components of unpleasant symptoms (67). CCL2 brings in monocytes and microglia and can change the way synapses function in limbic brain areas (19). This could make problems with social interaction and emotional expression worse.

These results are in line with what other studies have found. Brovcanin et al. found that higher levels of peripheral IL 17 were linked to higher total PANSS scores in chronic schizophrenia. This indirectly supports the idea that Th17 dysregulation is linked to more severe clinical symptoms (39). Also, Levraut et al. showed that CSF IL 17 levels are inversely related to negative symptom scales, which shows that Th17 cytokines have different patterns in the central and peripheral systems (68).

These results together suggest that activating the peripheral Th17 pathway may not only be a sign of systemic inflammation, but it may also be linked to the severity of negative symptoms in schizophrenia. These results show how important it is to study neuroimmune mechanisms and suggest that targeting Th17-related mediators might help with long-lasting and treatment-resistant negative symptoms. Longitudinal and mechanistic studies are still needed to prove causality and look at possible treatments.

### 4.6 Principal component analysis

The PCA of our investigation revealed distinct clustering of immune mediators. This indicates that inflammatory signaling in schizophrenia occurs in a systematic manner, according to organized, coordinated routes. The Th17 axis is crucial, as evidenced by the notable coloading of IL-21 and IL-23 on the main component. This corroborates prior findings indicating its involvement in persistent immunological activation and neuroinflammation associated with schizophrenia. The association of CCL2 with IL-22 likely illustrates the interplay between monocyte recruitment and tissue permeability. Both processes are frequently associated with issues pertaining to the blood–brain barrier and the activation of glial cells.

Although IL-21 and IL-23 did not differ significantly between patients and controls, both loaded strongly on one of the PCA-derived components. This likely reflects shared covariance with other Th17-related cytokines rather than an independent group effect. Importantly, our findings are consistent with the meta-analysis by Momtazmanesh et al. (11), which reported inconsistent case–control differences for these cytokines but highlighted their role within broader Th17 signaling networks. Therefore, the PCA results should be interpreted as hypothesis-generating, pointing to coordinated immune modules rather than stand-alone biomarkers (11).

The divergent loadings of CCL5 and CCL20 may signify regulatory vs active pathways for Th17 recruitment and the simultaneous production of IL-17 and IL-8 supports a conventional neutrophil-mediated inflammatory pattern. Other components of the immune system displayed atypical immune dynamics. The association of IL-8 and CXCL10 in opposing directions was particularly fascinating. This may suggest a change in the balance between the innate and adaptive immune systems, which would be noteworthy. The findings of our PCA demonstrate that schizophrenia is marked by a varied spectrum of immune responses. These findings suggest that specific cytokine–chemokine profiles may aid in the classification of subtypes or the identification of new biomarkers.

### 4.7 Strengths, limitations and future directions

One of the best things about this study is that it looks at Th17-related cytokines and key chemokines in schizophrenia patients at the same time. It also looks at how these things relate to symptom severity, laboratory indices and underlying immunological patterns using principal component analysis. An important strength of our protocol is that all blood samples were collected within the first 24 hours of hospital admission and prior to medication, thereby minimizing the confounding effects of treatment or prolonged hospitalization on cytokine levels. Using multiple layers of analysis gives us a three-dimensional view of immune dysregulation, which supports the idea that there are different inflammatory profiles in the disorder.

Nonetheless, specific issues require acknowledgment. The cross-sectional design complicates the determination of causality and precludes the observation of temporal variations in cytokine expression. It also leaves open the possibility of reverse causation, whereby chronic negative symptoms and related stressors may themselves drive immune activation. The study only examined a limited number of immune markers due to technical and financial limitations. Broader profiling, such as proteomics or transcriptomics, could reveal more relevant pathways. We can’t directly connect peripheral inflammation to central processes because we lack cerebrospinal fluid or neuroimaging data. The limited sample size implies that only large effect sizes could be detected with adequate power, while medium or small effects may have been overlooked. This underpowered nature is common in cytokine studies in schizophrenia, as highlighted by Klaus et al. (30), who also noted that their findings were restricted to large effects. Consequently, our results should be regarded as exploratory and require replication in larger cohorts. As a result, some true associations may have gone undetected. We therefore interpret our findings with caution, emphasizing the exploratory nature of this study.

To better understand how immune dysfunction affects schizophrenia pathophysiology and treatment response, future studies should use longitudinal sampling, multimodal biomarker integration and stratification by clinical subtype or treatment status.

## 5 Conclusion

This study provides evidence that the Th17 cytokine axis and associated chemokines are involved in the pathophysiology of schizophrenia. The elevation of IL-17, IL-22, IL-8, and CCL20 levels in patients, along with modest correlations between Th17-related markers and the severity of negative symptoms, suggests that a diverse range of immune system responses accompanies the illness. Principal component analysis further indicated that cytokine–chemokine alterations are not random but cluster into specific functional patterns. These findings imply that immune profiling may help identify clinically relevant subgroups and could inform the development of more effective anti-inflammatory interventions. Nevertheless, the weak associations with laboratory indices and the cross-sectional design underscore the need for long-term, multimodal studies to clarify temporal dynamics and to determine whether immune changes represent causes, consequences, or epiphenomena of schizophrenia.

## Data Availability

The raw data supporting the conclusions of this article will be made available by the authors, without undue reservation.
